# Hybrid Particle Swarm Optimization for Hybrid Flowshop Scheduling Problem with Maintenance Activities

**DOI:** 10.1155/2014/596850

**Published:** 2014-04-29

**Authors:** Jun-qing Li, Quan-ke Pan, Kun Mao

**Affiliations:** ^1^State Key Laboratory of Synthetic Automation for Process Industries, Northeastern University, Shenyang 110819, China; ^2^College of Computer Science, Liaocheng University, Liaocheng 252059, China

## Abstract

A hybrid algorithm which combines particle swarm optimization (PSO) and iterated local search (ILS) is proposed for solving the hybrid flowshop scheduling (HFS) problem with preventive maintenance (PM) activities. In the proposed algorithm, different crossover operators and mutation operators are investigated. In addition, an efficient multiple insert mutation operator is developed for enhancing the searching ability of the algorithm. Furthermore, an ILS-based local search procedure is embedded in the algorithm to improve the exploitation ability of the proposed algorithm. The detailed experimental parameter for the canonical PSO is tuning. The proposed algorithm is tested on the variation of 77 Carlier and Néron's benchmark problems. Detailed comparisons with the present efficient algorithms, including hGA, ILS, PSO, and IG, verify the efficiency and effectiveness of the proposed algorithm.

## 1. Introduction

The hybrid flowshop scheduling (HFS) problem has been researched by more and more literatures during last decades. HFS is a typical version of the flowshop scheduling problem (FSP), which has been proved to be an NP-hard problem. Therefore, HFS is also an NP-hard problem and has been researched by more and more heuristics or metaheuristics [1–11]. In the most present literature about HFS, the common situation is assumed that all machines are available in the production horizon. However, for some critical factors, such as machine random breakdown and preventive maintenance (PM) activity, machines are not available during the whole production horizon. Allaoui and Artiba solved the HFS with maintenance constraints by using an integrating simulation and optimization [12]. Xie and Wang discussed the complexity and algorithms for two-stage flexible flowshop scheduling with availability constraints [13]. Allaoui and Artiba again considered the two-stage HFS with maintenance constraints [14]. Ruiz et al. considered scheduling and preventive maintenance in the flowshop sequencing problem [15]. Naderi et al. applied variable neighborhood structure (VNS) algorithm for solving flexible flow line problems with sequence dependent setup times and different preventive maintenance policies [16]. Berrichi et al. presented a biobjective optimization algorithm for joint production and maintenance scheduling in the parallel machine environments [17]. Luo et al. developed a genetic algorithm for solving two-stage HFS with blocking and machine availability [18]. Allaoui and Artiba investigated Johnson's algorithm for solving optimally or approximately flowshop scheduling problems with unavailability periods [19]. Jabbarizadeh et al. developed a hybrid algorithm for solving the hybrid flexible flowshops with sequence-dependent setup times and machine availability constraints [20]. Besbes et al. tackled hybrid flowshop problem with nonfixed availability constraints [21]. Ma et al. gave a survey of scheduling with deterministic machine availability constraints [22]. Luo et al. solved the HFS with batch-discrete processors and machine maintenance in time windows [23]. Safari and Sadjadi tackled the flowshop scheduling problem with condition-based maintenance constraint and machines breakdown through a hybrid method [24]. Wang and Liu solved the two-stage hybrid flowshop scheduling with preventive maintenance using multiobjective tabu search method [25]. Rabiee et al. developed an intelligent hybrid metaheuristic for solving a case of no-wait two-stage flexible flowshop scheduling problem with unrelated parallel machines [26]. Allaoui and Artiba surveyed the maintenance constraints in HFS scheduling problems [27].

In this study, we developed a hybrid algorithm combining particle swarm optimization (PSO) and iterated local search (ILS) algorithms for solving the hybrid flowshop scheduling problems with PM activity. The rest of this paper is organized as follows: Section 2 briefly describes the problem. Next, the related algorithms are presented in Section 3.  Section 4 reports the framework of the proposed algorithm.  Section 5 illustrates the experimental results and compares them to the present performing algorithms from the literature to demonstrate the superiority of the proposed algorithm. Finally, the last section gives the concluding remarks and future research directions.

## 2. Problem Definition

In this study, we consider a hybrid flowshop scheduling problem in reality production system. The PM activity is considered in the considered HFS problems. Firstly, we give the following assumptions.

1. Each machine can process only one operation at a time, while each operation can be processed by only one machine at a time.
2. Preemption is not allowable; that is, each operation must be completed without interruption before its completion.
3. At each stage, more than one machine from identical parallel machines can be selected for each operation.
4. The processing time for each operation at each stage is determined.

Under the above assumption, the mathematical model for the problem is given as follows.

### 2.1. Variables

 
*i*: job index, *i* = 1,2,…, *n*,
 
*j*: stage index, *j* = 1,2,…, *s*,
 
*k*: machine index, *k* = 1,2,…, *m*,
 
*p*_*ij*_: the processing time of job *i* at stage *j*,
 
*s*_*i*,*j*_: the starting time of job *i* at stage *j*,
 
*c*_*i*,*j*_: the completion time of job *i* at stage *j*,
 
$\bar{s_{i,j}}$: the starting time of job *i* at stage *j* considering the PM activity,
 
$\bar{c_{i,j}}$: the completion time of job *i* at stage *j* considering the PM activity,
 
*PM*_*s*_^*k*^: the starting time point of the PM activity on *M*_*k*_,
 
*PM*_*e*_^*k*^: the completion time point of the PM activity on *M*_*k*_:
  (1) $$\begin{array}{c} Z_{ijk}=\{\begin{array}{cc} 1, & \text{if}\;\text{machine}\;k\;\text{is}\;\text{selected}\;\text{to}\;\text{process}\;\text{job}\;i\;\\ & \text{at}\;\text{stage}\;j\\ 0, & \text{otherwise}, \end{array}\\ Y_{ijk}=\{\begin{array}{cc} 1, & \text{if}\;s_{ij}\in \left[PM_{s}^{k},PM_{e}^{k}\right]\wedge Z_{ijk}=1\\ 0, & \text{otherwise}. \end{array} \end{array}$$

### 2.2. Problem Formula

(2) $$\begin{array}{c} f=\min\left\{\underset{1\leq i\leq n}{\max}c_{i,m}\right\} \end{array}$$

s.t.

(3) $$\begin{array}{c} \bar{c_{i,j}}\geq \bar{s_{i,j}}+\bar{p_{i,j}}+Y_{ijk}\left(PM_{e}^{k}-PM_{s}^{k}\right), \end{array}$$

(4) $$\begin{array}{c} \bar{s_{i+1,j}}\geq \bar{s_{i,j}}+\bar{p_{i,j}}, \end{array}$$

(5) $$\begin{array}{c} \bar{s_{i+1,j+1}}\geq \bar{c_{i,j}}, \end{array}$$

(6) $$\begin{array}{c} \underset{1\leq k\leq m}{\sum }Z_{ijk}=1,\;\forall i,j, \end{array}$$

(7) $$\begin{array}{c} Z_{ijk}=\left\{0,1\right\}\;\forall i,j,k, \end{array}$$

In the mathematical model, the objective is given in formula (2). Constraint (3) guarantees that the PM time should be considered in processing any operation. In Constraint (4), the operation sequence is realized for the same job; that is, the following operation cannot be started until the completion of the predecessor operation of the same job. Constraint (5) shows that, on the same machine, the following operation must wait for the completion of the predecessor operation. Constraint (6) guarantees that each job can select only one available machine at each stage.

## 3. The Related Algorithm

In this study, we consider combining PSO and ILS to construct a hybrid algorithm for solving the HFS with PM activity. The following is to illustrate the literature review of the two related algorithms.

### 3.1. ILS Algorithm

Iterated local search (ILS), firstly proposed by Stützle [28], is a metaheuristic to increase the ability to jump out of the local optima for the canonical local search methods. It has attracted much attention of researchers for its simplicity, effectiveness, and efficiency, and it has been applied successfully to traveling salesman problem, flowshop scheduling problem, job shop scheduling problem, and vehicle scheduling problem, [28–31] during recent years. The main frame of the canonical ILS is as follows.

**Step 1**

Generate an initial solution *x*; let *x*′ = *x* and x* = *x*.

**Step 2**

Generate a certain number of neighboring solutions around the given solution *x*′, find the best neighboring solution *x*′′, and update the best solution found so far.

**Step 3**

Let *x* = Accept(*x*′′, *x*).

**Step 4**

If the stop condition is not satisfied, generated *x*′ = perturb(*x*), go back to Step 2; otherwise, stop the algorithm.

### 3.2. Particle Swarm Optimization

In 1995, mimicking the flying behavior of a swarm of birds, a novel optimization algorithm named particle swarm optimization (PSO) was developed by Kennedy and Eberhart, which has been verified efficient for solving both continuous and discrete optimization problems [32]. During recent years, many researchers have applied PSO for solving lots of optimization problems [33–43].

The flowchart of the canonical PSO is given as follows.

**Step 1**

Set the system parameters, such as the initial population size, the possibility (*p*_*l*_) for learning from local best, and the possibility (*p*_*g*_) for learning from the best solution found so far.

**Step 2**

Generate the initial population of particles.

**Step 3**

Store each particle into a vector named local best, where each solution corresponds to the local best of the corresponding particle. Memorize the best solution found so far.

**Step 4**

For each particle, perform the following steps until the stop condition is satisfied.

**Step 5**

Randomly generate a number *r*_1_ between 0 and 1, if *r*_1_ is less than *p*_*l*_, and then perform the learning process from the local best of the current particle.

**Step 6**

Randomly generate a number *r*_1_ between 0 and 1, if *r*_1_ is less than *p*_*g*_, and then perform the learning process from the global best of the current particle.

**Step 7**

Record the local best for each particle and the global best found so far.

**Step 8**

Learn by itself.

**Step 9**

Go back to Step 4.

## 4. Framework of the Proposed Algorithm

### 4.1. Solution Representation

**Figure 1:**
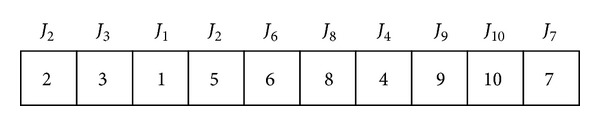
Solution representation.

For solving the HFS scheduling problems with PM activity, we use the permutation representation mechanism. Give a HFS scheduling problem *n* jobs, *s* stages, and *m* machines; each solution is represented by a vector of integer values, where each integer value represents a job number. Therefore, the length of the solution equals the number of jobs. For example, for a HFS problem with ten jobs and three stages, Figure 1 gives one solution representation, where the scheduling sequence is *J*_2_, *J*_3_, …, and *J*_7_.

The sequence in Figure 1 is only for the first stage; that is, at the first stage, each job is scheduled according to the above sequence, while for the following stages, the decoding mechanism is given as follows.

### 4.2. Decoding without Disruption

It can be seen from the solution representation that the machine selection is not included in the solution representation. The decoding for the above solution representation is given as follows.

**Step 1**

For the first stage, each job is scheduled according to their sequence in the solution representation. In Figure 1 the first job to be scheduled is *J*_2_ and the last one is *J*_7_. Each job selects the first available machine.

**Step 2**

In the following stages, each job is to be scheduled just after its completion of the previous stage, and select the first available machine from the candidate machines.

### 4.3. Decoding with PM Activity

**Figure 2:**
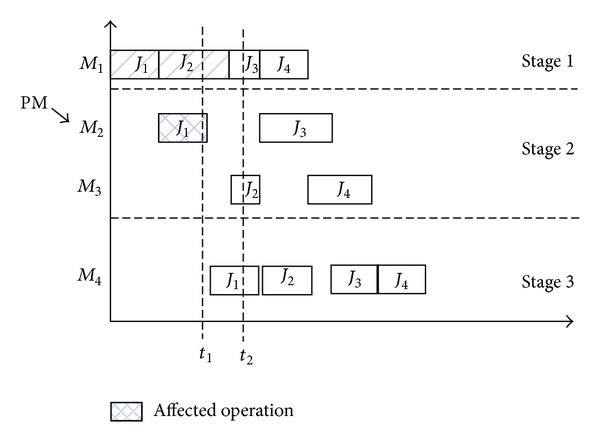
Situation 1 of PM activity.

When considering the PM activity, that is, at time *t*, there is a PM activity occurring on a given machine *M*_*k*_. Then two situations we should consider, that is, the first is that when an operation is just being processed on *M*_*k*_ when the disruption event occurs. The second situation is that the affected machine *M*_*k*_ is idle and no operation is affected by the PM activity.

(1)* Situation 1*. For the first situation, an operation is affected by the PM activity.  Figure 2 gives the example chart for the situation. From Figure 2, we can see that, at time point *t*_1_, the machine *M*_2_ shows a PM activity. It will restart its work at time point *t*_2_. However, before the PM activity of the machine, the operation *J*_1_ has started its work and cannot complete its work at time point *t*_1_. In this situation, we have to do the following works for different realistic production systems.

- When an operation is being processed and the processing machine needs to be maintenanced, we have to drop the affected operation and all its following operations. This is appliable for some certain realistic production system, such as steelmaking-casting system. Because of temperature restriction, an operation cannot wait for the restart work of the machine and has to be erased from the system because of its temperature loss. For example, for iron body, when its temperature decreases, its component structure will be destroyed.
- In another situation, the affected operation will keep its previous work and wait for the restart of the affected machine. When the affected machine is available, the affected operation can restart its work and continue the following work.

(2)* Situation 2*. For the second situation, no working operation is affected by the PM activity. In this situation, we should consider whether there is any operation which is allocated to the affected machine during the PM activity. That is, if an operation is scheduled to be processed on the affected machine before its restart, then we should reconsider the assignment rule, which is given as follows.

**Figure 3:**
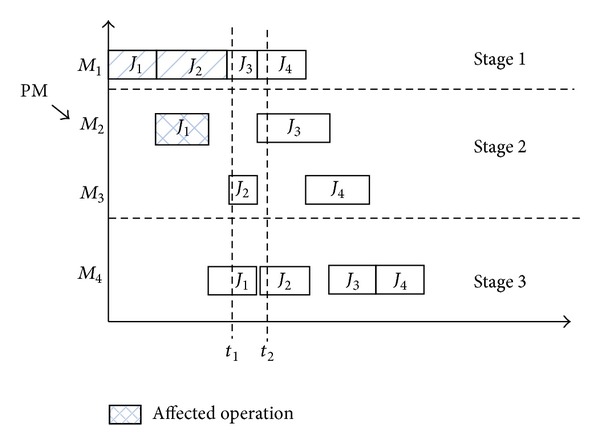
Situation 2 of PM activity.

- If an operation is scheduled to be processed on the affected machine, then the start time of the operation is located between the start and end time point of the PM activity. At that situation, we should assign a new machine for the affected operation if there is another available machine for the affected operation. For example, in Figure 3, the start time of the job *J*_3_ is between the start and end time of the PM event on *M*_2_. When the PM event occurs on the machine, we should assign another machine for *J*_3_; here, we can select *M*_3_ for processing *J*_3_.
- Another situation is that we cannot select another machine for the affected operation, because of the instability of the system. At that situation, we can only choose to keep the assignment machine for the affected operation and start its work after the availability of the affected machine.

### 4.4. Initialization Heuristic

In the initialization phase, we presented two heuristics, which are presented as follows.

(1)* The First Initial Heuristic*. The first initial heuristic is very simple and easy to implement, which is named INT-I with the following steps.

**Step 1**

Perform the following step for *P*_*s*_ times.

**Step 2**

Randomly generate a particle.

**Step 3**

Evaluate the new-generated particle and insert it into the current population.

(2)* The Second Initial Heuristic*. The second initial heuristic is named INT-II, which is given as follows.

**Step 1**

Generate a particle using the NEH approach [44] and insert it into the initial population.

**Step 2**

Perform the following step for *P*_*s*_ − 1 times.

**Step 3**

Randomly generate a particle and evaluate the new-generated particle.

**Step 4**

If the new-generated particle is not equal with any individual in the current population, then insert it into the initial population; otherwise, ignore it.

### 4.5. Discrete PSO Process

Each particle in the current population updates its status through the following three procedures: (1) learning through its history status, (2) learning through its local best, and (3) learning through the global best found so far.

Similar to [34], the discrete version of PSO is realized as follows.

- For the process of learning through its history status, we embed the mutation operator in the PSO algorithm. The mutation operators include swap, insert, multiple swap [34], and multiple insert. The multiple insert operator is developed firstly in this study. The detailed steps are as follows. Firstly, randomly produce a position *r*_1_ range at [2, *l*_en_ − 1], *c*, where *l*_en_ represents the length of the solution. Secondly, insert the element in the position (*r*_1_ − 1) to the position at (*r*_1_ + 1). Thirdly, evaluate the new-generated solution and replace the current solution if a better individual is found.
- For the process of learning through its local best and learning through the global best, apply the crossover operator between the two selected solutions. The detailed implementation of the crossover operators is discussed in the following section.

### 4.6. Crossover Operators

In [45], the authors verified many crossover operators for the regular flowshop (PMX or partially mapped crossover, OP or one point order crossover, TP or two-point order crossover, OX or order crossover, UOB or uniform order based, and several others). The results showed that the offspring generated after crossover tended to be worse than their progenitors on many occasions. In this study, we tested the following crossover operators in HFS with PM environments:

- PMX or partially mapped crossover;
- OP or one point order crossover;
- TP or two-point order crossover;
- PTL crossover [34].

### 4.7. ILS-Based Local Search

To further improve the searching ability of the proposed algorithm, we apply the ILS-based local search for the best solution found so far in each iteration. That is, after the three learning processes discussed in the above section, the ILS-based local search will be applied for the best solution for enhanced searching. The detailed steps of the ILS-based local search are given as follows.

**Step 1**

For the best solution, perform the following steps until the stop condition is satisfied.

**Step 2**

Destruction phase: randomly generate aposition in the current solution. Delete the corresponding element from the current solution.

**Step 3**

Construction phase: for the deleted element, perform the following steps.

*Step 3* *.1*. For each candidate position in the current solution, insert the deleted element and evaluate the partial solution.

*Step 3.2*. Select the best position for the deleted element and insert it into the best position.

### 4.8. Framework of the Proposed Algorithm

In this study, we proposed a hybrid algorithm for solving the HFS problem with PM activity. In the decoding procedure, we select the following rules to decode each solution; in Situation 1, we choose to keep the work of the affected operation and continue its work after the affected machine is available. In Situation 2, we choose to assign another machine for the affected operation.

The flowchart of the proposed algorithm is given as follows.

**Step 1**

Set the system parameters.

**Step 2**

Produce the initial population of particles.

**Step 3**

Evaluate each particle and record the best solution found so far.

**Step 4**

If the stop condition is satisfied, stop the algorithm. Otherwise, perform the following steps.

**Step 5**

Perform learning phase.

*Step 5.1*. Perform the procedure of learning by itself.

*Step 5.2*. Perform the procedure of learning through its local best.

*Step 5.3*. Perform the procedure of learning through the global best.

**Step 6**

ILS-based local search phase: for the best solution found so far, perform the ILS-based local search procedure.

**Step 7**

Go back to Step 4.

## 5. Numerical Analysis

The proposed algorithm is coded in C++, on DELL i7 CPU with 16 GB memory. For each instance, we conduct 20 independently runs, and the best, worst, and average values are collected for comparisons.

### 5.1. Experimental Data

The proposed PSO-ILS algorithm was tested using the variation of the benchmark problems provided by Carlier and Néron [46]. There are 77 instances in Carlier and Néron's benchmark problems, which range from 10 jobs and 5 stages to 15 jobs and 10 stages. Each instance is represented by a three-number file name. The three numbers are number of jobs, number of stages, and problem structure index, which can be referred in [46]. For simplicity, the variations of the 77 benchmark problems are set with the same name. The variation implementation is implemented as follows.

- For each instance, run the proposed algorithm without considering any PM activity and get the baseline result.
- In each baseline result, at each stage, randomly select a time point *t* at which a machine (hereafter called *m*_*k*_) is working.
- Select the working machine (*m*_*k*_) and generate a random PM activity duration *d*_*b*_.
- Record the PM activity data, including the PM time window [*t*, *t* + *d*_*b*_], and the affected machine *m*_*k*_.

### 5.2. Parameter Tuning for PSO

In the canonical PSO algorithm, the parameters are as follows:

- population size: *P*_*s*_;
- learning probability from the local best: *c*_1_;
- learning probability from the global best: *c*_2_;
- learning probability by itself: *p*_*m*_;
- crossover operator type;
- mutation operator type.

For each instance, we memorized the best solution found by all the compared algorithms and calculated the relative percentage deviation over the best solution for each compared algorithm, which is computed as follows:

(8) $$\begin{array}{c} {\text{RPD}}_{i}=\frac{{\text{Comp}}_{i}^{k}-{\text{Best}}_{i}}{{\text{Best}}_{i}}\times 100, \end{array}$$

where Comp_*i*_^*k*^ is the optimal solution found by the *k*th compared algorithm, while Best_*i*_ is the best solution found by all the compared algorithms. In the comparison results, we just calculated the average relative percentage deviation ($\bar{\text{RPD}}$) for each instance.

#### 5.2.1. Crossover Type

To test the impact of different crossover operators, we implemented five kinds of crossover operators, that is, one-point crossover (OP), two-point crossover (TP), partially mapped crossover (PMX), similar job 2-point crossover (SJ2OX), and PTL crossover operator [34]. The description of the given crossover operators is given in Table 1. The comparisons results of different crossover types are given in Table 2. In Table 2, the instance name is given in the first column, while the following five columns report the $\bar{\text{RPD}}$ values for the five compared algorithms. From the results we can see that (1) the algorithm with PTL crossover operator gets better values for 75 out of 77 instances, except for the two instances, that is, Case 13 and Case 22; (2) for solving the given 77 instances with PM activity, in average, the algorithm with PTL crossover operator obtains a relative better result, which is obviously better than the other four compared algorithms. The following algorithms are SJ2OX, TP, PMX, and OP, respectively.

#### 5.2.2. Crossover Probability

The crossover probability for learning from the local best (*c*_1_) and the learning probability from the global best (*c*_2_) are critical for the algorithm. In order to test different learning probabilities, we test five kinds of probabilities, which are given in Table 3. The comparison results for different learning probability are given in Table 4. It can be seen from Table 4 that CP-I is the best among the five compared algorithms. That is, the two crossover probabilities *c*_1_ and *c*_2_ are set to 0.2 and 0.2, respectively.

#### 5.2.3. Mutation Type

To test the impact of different mutation operators, we implemented four kinds of mutation operators, that is, the swap, insert, multiple swap, and multiple insert operators, which are given in Table 5.  Table 6 gives the comparison results of different mutation types. It can be seen from Table 6 that the proposed multiple insert mutation operator performs the best among the compared algorithms.

#### 5.2.4. Mutation Probability

To test the impact of different mutation probabilities, we implemented five kinds of mutation probabilities, that is, 0.1, 0.2, 0.5, 0.8, and 0.9, which are given in Table 7.  Table 8 gives the comparison results of different mutation probabilities. It can be seen from Table 8 that mutation probability with the value 0.9 performs the best among the compared algorithms.

#### 5.2.5. Population Size

To test the impact of different population sizes, we implemented five kinds of population sizes, that is, 10, 20, 30, 50, and 100, which are given in Table 9.  Table 10 gives the comparison results of different population sizes. It can be seen from Table 10 that population size with the value 100 performs the best among the compared algorithms.

#### 5.2.6. The Final Parameters

After the comparison results for each kind of parameter, we can conclude the best parameters for the canonical PSO algorithm, which are given in Table 11.

### 5.3. Comparisons Analysis

To make a pair comparison with the present efficient algorithms, we coded the following algorithms to solve the HFS problem with PM activity. These compared algorithms include hGA by Ruiz and Maroto [47], IG by Ruiz and Stützle [48], ILS by Dong et al. [31], and PSO by Liao et al. [49]. The parameters for the compared algorithms are set to the same values in their literature, except that the stop condition is set to 20 seconds.

The comparison results for the best $\bar{\text{RPD}}$ values are given in Table 12. It can be seen from Table 12 that (1) for solving the HFS with PM activities, the proposed algorithm obtains all optimal results for 77 benchmark instances, which is obviously better than the other compared algorithms; (2) in average, the proposed algorithm is also better than the other compared algorithms; (3) the proposed PSO-ILS algorithm is better than the canonical PSO algorithm, which also verifies the efficiency of the ILS-based local search; (4) the proposed algorithm is better than the canonical IG algorithm, which shows the exploration ability of the proposed algorithm.

Table 13 reports the comparison results for the average $\bar{\text{RPD}}$ values. It can be seen from Table 13 that (1) the proposed algorithm obtains 74 optimal values out of 77 instances; (2) in average, the PSO-ILS algorithm obtains the best average $\bar{\text{RPD}}$ values, which is obviously better than the other algorithms. The following algorithms are PSO, hGA, ILS, and IG, respectively.

## 6. Conclusions

In this study, we proposed a hybrid algorithm for solving the HFS with PM activities. In the proposed algorithms, different crossover and mutation operators are applied for the learning procedure. The ILS-based local search procedure is embedded in the proposed algorithm to further improve the searching ability of the algorithm. Variation versions of 77 Carlier and Néron's benchmark problems are presented to adapt to the realistic industrial horizon. Experimental comparisons with four present algorithms show the efficiency and effectiveness of the proposed algorithm. The future work is to apply the proposed algorithm for solving rescheduling problems in hybrid and flexible environments [50–52].

## Figures and Tables

**Table 1:** Crossover type.

Crossover type	Description
CT-I	OP
CT-II	TP
CT-III	PMX
CT-IV	SJ2OX
CT-V	PTL

**Table 2:** Comparisons of different crossover types.

Case	$\overset{-}{\text{RPD}}$				
	CT-I	CT-II	CT-III	CT-IV	CT-V
j10c5a2	0.00	0.00	0.00	0.00	0.00
j10c5a3	0.00	0.00	0.00	0.00	0.00
j10c5a4	0.00	0.00	0.00	0.00	0.00
j10c5a5	0.00	0.00	0.00	0.00	0.00
j10c5a6	0.00	0.00	0.00	0.00	0.00
j10c5b1	0.00	0.00	0.00	0.00	0.00
j10c5b2	0.00	0.00	0.00	0.00	0.00
j10c5b3	0.00	0.00	0.00	0.00	0.00
j10c5b4	0.00	0.00	0.00	0.00	0.00
j10c5b5	0.00	0.00	0.00	0.00	0.00
j10c5b6	0.00	0.00	0.00	0.00	0.00
j10c5c1	0.00	0.00	0.00	0.00	0.00
j10c5c2	0.00	1.35	1.35	1.35	1.35
j10c5c3	0.00	0.00	0.00	0.00	0.00
j10c5c4	1.52	0.00	0.00	0.00	0.00
j10c5c5	0.00	0.00	0.00	0.00	0.00
j10c5c6	0.00	0.00	0.00	0.00	0.00
j10c5d1	0.00	0.00	0.00	0.00	0.00
j10c5d2	0.00	0.00	0.00	0.00	0.00
j10c5d3	0.00	0.00	0.00	0.00	0.00
j10c5d4	0.00	0.00	0.00	0.00	0.00
j10c5d5	0.00	1.52	3.03	1.52	1.52
j10c5d6	0.00	0.00	0.00	0.00	0.00
j10c10a1	0.00	0.00	0.00	0.00	0.00
j10c10a2	0.00	0.00	0.00	0.00	0.00
j10c10a3	0.00	0.00	0.00	0.00	0.00
j10c10a4	0.00	0.00	0.00	0.00	0.00
j10c10a5	0.00	0.00	0.00	0.00	0.00
j10c10a6	0.00	0.00	0.00	0.00	0.00
j10c10b1	0.00	0.00	0.00	0.00	0.00
j10c10b2	0.00	0.00	0.00	0.00	0.00
j10c10b3	0.00	0.00	0.00	0.00	0.00
j10c10b4	0.00	0.00	0.00	0.00	0.00
j10c10b5	0.00	0.00	0.00	0.00	0.00
j10c10b6	0.00	0.00	0.00	0.00	0.00
j10c10c1	0.00	0.00	0.00	0.00	0.00
j10c10c2	0.00	0.00	0.00	0.00	0.00
j10c10c3	0.86	0.00	0.00	0.00	0.00
j10c10c4	0.00	0.00	0.00	0.00	0.00
j10c10c5	0.00	0.00	0.00	0.00	0.00
j10c10c6	0.00	0.00	0.00	0.00	0.00
j15c5a1	0.00	0.00	0.00	0.00	0.00
j15c5a2	0.00	0.00	0.00	0.00	0.00
j15c5a3	0.00	0.00	0.00	0.00	0.00
j15c5a4	0.00	0.00	0.00	0.00	0.00
j15c5a5	0.00	0.00	0.00	0.00	0.00
j15c5a6	0.00	0.00	0.00	0.00	0.00
j15c5b1	0.00	0.00	0.00	0.00	0.00
j15c5b2	0.00	0.00	0.00	0.00	0.00
j15c5b3	0.00	0.00	0.00	0.00	0.00
j15c5b4	0.00	0.00	0.00	0.00	0.00
j15c5b5	0.00	0.00	0.00	0.00	0.00
j15c5b6	0.00	0.00	0.00	0.00	0.00
j15c5c1	1.18	1.18	0.00	1.18	0.00
j15c5c2	0.00	0.00	0.00	0.00	0.00
j15c5c3	1.15	0.00	1.15	0.00	0.00
j15c5c4	1.12	1.12	1.12	1.12	0.00
j15c5c5	2.67	0.00	0.00	0.00	0.00
j15c5c6	0.00	0.00	0.00	0.00	0.00
j15c5d1	0.00	0.00	0.00	0.00	0.00
j15c5d2	1.18	0.00	0.00	0.00	0.00
j15c5d3	0.00	0.00	0.00	0.00	0.00
j15c5d4	2.38	1.19	1.19	1.19	0.00
j15c5d5	0.00	1.25	1.25	0.00	0.00
j15c5d6	1.23	1.23	0.00	1.23	0.00
j15c10a1	0.00	0.00	0.00	0.00	0.00
j15c10a2	0.00	0.00	0.00	0.00	0.00
j15c10a3	0.00	0.00	0.00	0.00	0.00
j15c10a4	0.00	0.00	0.00	0.00	0.00
j15c10a5	0.00	0.00	0.00	0.00	0.00
j15c10a6	0.00	0.00	0.00	0.00	0.00
j15c10b1	0.00	0.00	0.00	0.00	0.00
j15c10b2	0.00	0.00	0.00	0.00	0.00
j15c10b3	0.00	0.00	0.00	0.00	0.00
j15c10b4	0.00	0.00	0.00	0.00	0.00
j15c10b5	0.00	0.00	0.00	0.00	0.00
j15c10b6	0.00	0.00	0.00	0.00	0.00

Average	0.17	0.11	0.12	0.10	0.04

**Table 3:** Crossover probability.

Crossover probability	*c*_ 1_	*c*_ 2_
CP-I	0.2	0.2
CP-II	0.2	0.8
CP-III	0.5	0.5
CP-IV	0.8	0.2
CP-V	0.8	0.8

**Table 4:** Comparisons of different crossover probabilities.

Case	$\overset{-}{\text{RPD}}$				
	CP-I	CP-II	CP-III	CP-IV	CP-V
Average	**0.08 **	0.20	0.15	0.13	0.11

**Table 5:** Mutation probability.

Mutation type	Description
MT-I	Swap
MT-II	Insert
MT-III	Multiple swap
MT-IV	Multiple insert

**Table 6:** Comparisons of different mutation types.

Case	$\overset{-}{\text{RPD}}$			
	MT-I	MT-II	MT-IIII	MT-IV
Average	0.08	0.08	0.02	**0.01**

**Table 7:** Mutation probability.

Mutation probability	*p*_*m*_
MP-I	0.1
MP-II	0.2
MP-III	0.5
MP-IV	0.8
MP-V	0.9

**Table 8:** Comparisons of different mutation types.

Case	$\overset{-}{\text{RPD}}$				
	MP-I	MP-II	MP-III	MP-IV	MP-V
Average	0.12	0.09	0.06	0.08	**0.02**

**Table 9:** Population size.

Population size	*p*_*s*_
PS-I	10
PS-II	20
PS-III	30
PS-IV	50
PS-V	100

**Table 10:** Comparisons of different population sizes.

Case	$\overset{-}{\text{RPD}}$				
	PS-I	PS-II	PS-III	PS-IV	PS-V
Average	0.06	0.08	0.03	0.06	**0.00 **

**Table 11:** Different parameters for the canonical PSO.

	Parameter	Value	Description
1	Crossover type	CT-V	PTL
2	Crossover probability	CP-I	*c*_1_ = 0.2, *c*_2_ = 0.2
3	Mutation type	MT-IV	Multiple insert
4	Mutation probability	MP-V	0.9
5	Population size	PS-V	100

**Table 12:** Comparisons of the best $\overset{-}{\text{RPD}}$ values.

Case	$\overset{-}{\text{RPD}}$				
	PSO-ILS	ILS	IG	PSO	hGA
j10c5a2	0.00	0.00	0.00	0.00	0.00
j10c5a3	0.00	0.00	0.00	0.00	0.00
j10c5a4	0.00	0.00	0.00	0.00	0.00
j10c5a5	0.00	0.00	0.00	0.00	0.00
j10c5a6	0.00	0.00	0.00	0.00	0.00
j10c5b1	0.00	0.00	0.00	0.00	0.00
j10c5b2	0.00	0.00	0.00	0.00	0.00
j10c5b3	0.00	0.00	0.00	0.00	0.00
j10c5b4	0.00	0.00	1.64	0.00	0.00
j10c5b5	0.00	0.00	0.00	0.00	0.00
j10c5b6	0.00	0.00	0.00	0.00	0.00
j10c5c1	0.00	7.35	5.88	0.00	0.00
j10c5c2	0.00	2.70	2.70	0.00	0.00
j10c5c3	0.00	4.17	2.78	0.00	0.00
j10c5c4	0.00	4.55	4.55	0.00	0.00
j10c5c5	0.00	5.13	1.28	0.00	0.00
j10c5c6	0.00	4.35	1.45	0.00	0.00
j10c5d1	0.00	4.55	1.52	0.00	0.00
j10c5d2	0.00	1.35	0.00	0.00	0.00
j10c5d3	0.00	3.13	1.56	0.00	0.00
j10c5d4	0.00	2.86	2.86	0.00	0.00
j10c5d5	0.00	4.55	4.55	1.52	1.52
j10c5d6	0.00	4.84	3.23	0.00	0.00
j10c10a1	0.00	0.00	0.00	0.00	0.00
j10c10a2	0.00	2.53	3.16	0.00	0.00
j10c10a3	0.00	1.35	0.00	0.00	0.00
j10c10a4	0.00	0.00	0.00	0.00	0.00
j10c10a5	0.00	0.00	0.00	0.00	0.00
j10c10a6	0.00	2.05	4.11	0.00	0.00
j10c10b1	0.00	0.00	0.00	0.00	0.00
j10c10b2	0.00	0.64	0.64	0.00	0.00
j10c10b3	0.00	0.00	0.00	0.00	0.00
j10c10b4	0.00	0.00	0.00	0.00	0.00
j10c10b5	0.00	0.00	0.00	0.00	0.00
j10c10b6	0.00	0.00	0.00	0.00	0.00
j10c10c1	0.00	2.61	1.74	0.00	0.00
j10c10c2	0.00	2.52	1.68	0.00	0.00
j10c10c3	0.00	3.45	2.59	0.00	0.00
j10c10c4	0.00	2.50	1.67	0.00	0.00
j10c10c5	0.00	1.59	3.17	0.00	0.00
j10c10c6	0.00	1.89	3.77	0.00	0.00
j15c5a1	0.00	0.00	0.00	0.00	0.00
j15c5a2	0.00	0.00	0.00	0.00	0.00
j15c5a3	0.00	0.00	0.00	0.00	0.00
j15c5a4	0.00	0.00	0.00	0.00	0.00
j15c5a5	0.00	0.00	0.00	0.00	0.00
j15c5a6	0.00	0.00	0.00	0.00	0.00
j15c5b1	0.00	0.00	0.00	0.00	0.00
j15c5b2	0.00	0.00	0.00	0.00	0.00
j15c5b3	0.00	0.00	0.00	0.00	0.00
j15c5b4	0.00	0.00	0.00	0.00	0.00
j15c5b5	0.00	0.60	0.00	0.00	0.00
j15c5b6	0.00	0.00	0.00	0.00	0.00
j15c5c1	0.00	5.88	8.24	0.00	1.18
j15c5c2	0.00	4.40	4.40	0.00	0.00
j15c5c3	0.00	10.34	8.05	0.00	0.00
j15c5c4	0.00	3.37	5.62	0.00	0.00
j15c5c5	0.00	9.46	10.81	0.00	1.35
j15c5c6	0.00	7.69	6.59	0.00	0.00
j15c5d1	0.00	0.00	0.00	0.00	0.00
j15c5d2	0.00	9.52	9.52	1.19	0.00
j15c5d3	0.00	7.23	6.02	0.00	0.00
j15c5d4	0.00	7.14	5.95	1.19	1.19
j15c5d5	0.00	8.86	8.86	1.27	0.00
j15c5d6	0.00	4.94	4.94	1.23	1.23
j15c10a1	0.00	0.00	0.00	0.00	0.00
j15c10a2	0.00	2.00	2.00	0.00	0.00
j15c10a3	0.00	0.00	1.01	0.00	0.00
j15c10a4	0.00	0.00	1.78	0.00	0.00
j15c10a5	0.00	0.55	0.00	0.00	0.00
j15c10a6	0.00	1.00	0.00	0.00	0.00
j15c10b1	0.00	0.00	0.00	0.00	0.00
j15c10b2	0.00	0.00	0.00	0.00	0.00
j15c10b3	0.00	0.00	0.00	0.00	0.00
j15c10b4	0.00	0.00	0.00	0.00	0.00
j15c10b5	0.00	0.00	0.00	0.00	0.00
j15c10b6	0.00	0.00	0.00	0.00	0.00

Average	0.00	2.00	1.82	0.08	0.08

**Table 13:** Comparisons of average $\bar{\text{RPD}}$ values.

Case	$\bar{\text{RPD}}$				
	PSO-ILS	ILS	IG	PSO	hGA
j10c5a2	0.00	0.00	0.00	0.00	0.00
j10c5a3	0.00	0.00	0.00	0.00	0.00
j10c5a4	0.00	0.00	0.00	0.00	0.00
j10c5a5	0.00	0.00	0.00	0.00	0.00
j10c5a6	0.00	0.73	1.45	0.00	0.00
j10c5b1	0.00	0.00	0.00	0.00	0.00
j10c5b2	0.00	0.00	0.00	0.00	0.00
j10c5b3	0.00	0.55	0.00	0.00	0.00
j10c5b4	0.00	0.49	3.44	0.00	0.00
j10c5b5	0.00	0.00	0.00	0.00	0.00
j10c5b6	0.00	0.00	0.00	0.00	0.00
j10c5c1	0.29	8.82	8.53	0.00	0.00
j10c5c2	0.81	3.50	3.77	0.00	0.00
j10c5c3	0.00	5.83	4.17	0.00	0.00
j10c5c4	0.00	6.36	6.06	0.00	0.00
j10c5c5	0.00	6.41	3.59	0.00	0.00
j10c5c6	0.00	5.80	4.93	0.00	0.00
j10c5d1	0.00	5.15	4.85	0.00	0.00
j10c5d2	0.00	2.97	2.70	0.00	0.00
j10c5d3	0.00	6.25	5.94	0.00	0.00
j10c5d4	0.00	3.71	4.57	0.00	0.00
j10c5d5	0.00	5.11	5.11	0.60	0.60
j10c5d6	0.00	6.45	5.81	0.00	0.00
j10c10a1	0.00	0.00	0.00	0.00	0.00
j10c10a2	0.00	3.67	3.67	0.00	0.00
j10c10a3	0.00	2.03	0.68	0.00	0.00
j10c10a4	0.00	0.00	1.48	0.00	0.00
j10c10a5	0.00	0.00	3.24	0.00	0.00
j10c10a6	0.00	3.70	4.79	0.00	0.00
j10c10b1	0.00	0.00	0.00	0.00	0.00
j10c10b2	0.00	0.89	2.80	0.00	0.00
j10c10b3	0.00	0.12	0.00	0.00	0.00
j10c10b4	0.00	0.00	0.00	0.00	0.00
j10c10b5	0.00	0.00	0.12	0.00	0.00
j10c10b6	0.00	0.36	0.36	0.00	0.00
j10c10c1	0.00	3.83	3.48	0.00	0.00
j10c10c2	0.00	3.53	2.69	0.00	0.00
j10c10c3	0.00	3.61	3.09	0.17	0.34
j10c10c4	0.00	3.17	2.83	0.00	0.00
j10c10c5	0.00	4.92	5.71	0.00	0.00
j10c10c6	0.00	2.45	4.53	0.00	0.00
j15c5a1	0.00	0.79	0.56	0.00	0.00
j15c5a2	0.00	0.00	0.00	0.00	0.00
j15c5a3	0.00	0.00	0.00	0.00	0.00
j15c5a4	0.00	0.00	0.26	0.00	0.00
j15c5a5	0.00	0.00	0.49	0.00	0.00
j15c5a6	0.00	0.00	0.00	0.00	0.00
j15c5b1	0.00	0.00	0.00	0.00	0.00
j15c5b2	0.00	0.00	0.00	0.00	0.00
j15c5b3	0.00	0.00	0.13	0.00	0.00
j15c5b4	0.00	0.54	0.68	0.00	0.00
j15c5b5	0.00	1.93	1.08	0.00	0.00
j15c5b6	0.00	0.00	0.11	0.00	0.00
j15c5c1	0.00	7.71	8.88	0.23	0.47
j15c5c2	0.00	6.81	6.59	0.22	0.00
j15c5c3	0.00	11.72	10.11	0.23	0.23
j15c5c4	0.00	5.58	5.80	0.22	0.00
j15c5c5	0.00	10.46	12.06	0.80	1.88
j15c5c6	0.00	9.45	9.01	0.00	0.00
j15c5d1	0.00	0.00	0.00	0.00	0.00
j15c5d2	0.00	9.48	9.48	0.95	0.47
j15c5d3	0.00	7.93	7.45	0.24	0.00
j15c5d4	0.00	7.58	7.58	0.71	0.71
j15c5d5	0.25	10.53	10.53	0.25	0.00
j15c5d6	0.00	6.14	6.39	0.74	0.74
j15c10a1	0.00	0.17	0.00	0.00	0.00
j15c10a2	0.00	3.30	3.20	0.00	0.00
j15c10a3	0.00	0.61	2.32	0.00	0.00
j15c10a4	0.00	0.00	1.96	0.00	0.00
j15c10a5	0.00	0.77	0.77	0.00	0.00
j15c10a6	0.00	2.70	0.10	0.00	0.00
j15c10b1	0.00	0.00	0.00	0.00	0.00
j15c10b2	0.00	0.00	0.21	0.00	0.00
j15c10b3	0.00	0.00	0.00	0.00	0.00
j15c10b4	0.00	0.00	0.00	0.00	0.00
j15c10b5	0.00	0.60	0.20	0.00	0.00
j15c10b6	0.00	0.00	0.00	0.00	0.00

Average	0.02	2.67	2.73	0.07	0.07
